# How can you be a doctor? Ableism in the workplace

**DOI:** 10.4102/ajod.v14i0.1588

**Published:** 2025-03-31

**Authors:** Sarah N. Whitehead, Seyi L. Amosun, Theresa Lorenzo, Harsha Kathard

**Affiliations:** 1 Department of Health Sciences Education, Faculty of Health Sciences, University of Cape Town, Cape Town, South Africa; 2 Department of Health and Rehabilitation Sciences, Faculty of Health Sciences, University of Cape Town, Cape Town, South Africa

## Background

Meeting the challenges of disability inclusion is the collective responsibility of society, including medical doctors (Battalova et al. 2020; Lindsay et al. 2023; Thomson & Murray 2023). The shift to a more diverse workforce that includes physicians with disabilities is gaining considerable international traction (Singh & Meeks 2023), and communities are becoming receptive to engaging with medical doctors with disabilities (Jarus et al. 2020; Mogensen & Hu 2019). To strengthen these achievements, the philosophy of disability inclusion must be adjusted from one where students and practitioners with disabilities are viewed as problematic and having to ‘overcome’ disability to one where institutions anticipate and welcome them in recognition of a diverse community (Fitzpatrick & Barrett 2023; Singh & Meeks 2023).

Medical professionals with disabilities still face challenges in the workplace (Jarus et al. 2023; Lindsay et al. 2023; Rimmer 2020), and some medical associations have taken steps to ensure appropriate accommodation is provided. For example, the Canadian Society of Physician Leaders (Munro, Quon & Gartke 2021), the American Medical Association (Waliany 2016), the British Medical Association (Rimmer 2020) and the General Medical Council of the United Kingdom (Mogensen & Hu 2019) are developing principles that value these practitioners with recommendations that promote their reasonable accommodation and provide them with equitable opportunities.

The South African Medical Association (SAMA) represents the interests of medical doctors, yet how this commitment enhances the provision of reasonable accommodation to their members with disabilities is unclear (Suich & Schneider 2022). ‘Is ableism still entrenched in the medical profession in South Africa?’ (Whitehead et al. 2024) is the title of an article in which Dr Whitehead (a qualified and registered medical practitioner) described some of her experiences as a student and a practitioner. This publication triggered an invitation to present at an ethics conference for medical practitioners in South Africa, hoping to raise awareness about disability among fellow medical practitioners.

## Conference presentation

In this opinion paper the authors share the presentation prepared by Dr Whitehead for the Ethics Symposium, held at the Centre for Diabetes and Endocrinology on 23 August 2024, Johannesburg to assist SAMA and the general society in understanding and becoming more aware of disability.

When I was first asked to talk today, I knew it was a huge honour to be asked but I was not sure it was something I could practically do. I took some time to think about it. I realised it was a golden opportunity to raise awareness about disability inclusion and hopefully cause some shifts in the behaviour and attitudes of those of you here today. So here I am, ready to give this a go.

As a person with a disability, I think that in many instances members of the medical profession struggle in interactions with patients with disability. As a doctor with a disability, though aware that doctors may feel overwhelmed by the demands of practising medicine in general (Lagu et al. 2022), I feel that the medical profession still fails to maintain an unbiased and non-judgmental attitude towards colleagues with disabilities. These additional demands include improving the education of clinicians about the care of persons with disabilities and removing structural barriers in the health care delivery system. I do not make these statements lightly. I have plenty of lived experience to legitimise my concerns about my profession.

Whilst I was doing my PhD, I came across multiple pieces of evidence emphasising that Medicine, at its core, is meant to be a caring profession devoid of bias and judgment towards the members of society with whom the profession interacts (Trzeciak, Mazzarelli & Booker 2019). As a doctor, I believe that I have a right to be very honest about my profession. As professionals, we generally do well in following the ideals mentioned by Trzeciak et al (2019) when it comes to our interactions with most of our patients. I am aware that some doctors try to practise disability-inclusive medicine. I also know that in almost all of the cases where doctors fail to practise disability-inclusive medicine with both patients and colleagues with disabilities, there is no malicious intent. Their inability to be fully inclusive stems more from a lack of awareness and from deep-seated unconscious bias which is rooted in a phenomenon termed Ableism.

Ableism is the discrimination of and social prejudice against people with disabilities based on the belief that able-bodied people are superior, that disabled people require ‘fixing’, and defines people by their disability (Dekker 2022; Lindsay et al. 2023). As a person with a disability, I have experienced my fair share of ableism from many people within society but perhaps the harshest forms of ableism that I have encountered have come from within the medical profession.

I want to again stress that this is not meant to be a pity party, nor am I setting out to unfairly lambast the profession. Being on the receiving end of ableism is tough and can be hurtful but as I have said, most ableist behaviour is not done with any conscious malice. Through sharing some of my experiences, I’m trying to raise everyone’s conscious awareness about such behaviour and the negative impact it can have.

I have been a doctor for 14 years and I have never had to deal with the exact words of the question in the title of my talk, ‘How can you be a doctor?’ I have however had to manage situations where my ability to be a doctor has been questioned in other ways.

One such situation happened roughly 7 years after I qualified. I remember walking into the ward of the rehab unit where I worked. I was using my walker and had my stethoscope around my neck. There was a man with one of my rehab patients who I had never met but I heard my patient call him a doctor. This doctor saw me talking to a nurse, came towards me, put his hands on my stethoscope that was around my neck, and said ‘What are you doing with this?’

When that doctor grabbed my stethoscope and asked what I was doing with it, he immediately defined me by the disability he saw - the walking challenges and that I was using a walker. He was essentially placing me in a box with one label of disability on it and saying that I belong in the confines of that box only. His actions neglected to take into account that my disability is just one aspect of the multiple aspects that make up the person that I am.

I would love to say that I fired off a witty and sharp response to this question, but the reality is that I was completely shocked and speechless. I managed to get out, ‘I am a doctor’. I kept my emotions in check until he left my ward and then I collapsed in tears. I remember thinking, ‘Even if I was a confused patient who had somehow found a stethoscope and thought that I was a doctor, you do not treat another human being like that’.

It wasn’t until a few years after this incident when I was writing my PhD thesis, that I developed the skills and vocabulary to consider the impact of this behaviour and the other forms of ableism in the workplace that I have experienced. I think I am quite a mentally and emotionally strong person, but that incident did knock my confidence in being a doctor. I’m incredibly lucky to have established a wonderful support structure, that affirmed my being a doctor. My rehab medicine doctor colleague defended me in an email to this doctor - who questioned my identity in this rather inhumane manner - saying amongst other things, ‘Don’t forget that Dr Whitehead earned her medical degree in the same way you did!’ The e-mail statement speaks to the belief that many people have about disability. The belief is that if a disability is present - regardless of the type - then the individual must be intellectually challenged as well.

This belief along with other forms of ableist behaviour is based on predetermined beliefs and assumptions about disability. The limiting effects of ableism are bidirectional. It limits the expression of the identity of the person with a disability beyond their disability, but it is also limiting to the person enacting the ableism, in terms of interpersonal growth and introspection.

Medical professionals deal with human beings from all walks of life, which includes people with disabilities. My PhD research showed that there is a global call for healthcare to find ways to ensure better inclusion of people with disabilities and to afford them quality healthcare. This inclusion should not be limited to patients with disabilities. That in itself is ableism. We should be actively seeking out ways to include people with disabilities as our medical professional colleagues as well.

In closing, if you focus only on the disability, you become blind to the potential abilities of the individual with the disability. In my case, I have the same theoretical knowledge that a medical degree gives any graduate, but I have a lived experience of disability which sets me apart from many of my medical colleagues. This lived experience allows me to develop a quick, honest and very real rapport with my patients. Recognition and celebration of this ability by my physical rehabilitation medicine colleagues helped me find a field of medicine where I felt accepted and valued. So, I ask that you become more aware of your behaviour towards people with disabilities. Don’t be so quick to judge or assume anything. Take a bit of time to get to know the person with a disability and their abilities. Don’t define them based on their disability. Rather encourage and allow their abilities to define them.

## Conclusions

This opinion paper describes Dr Whitehead’s experiences as a medical practitioner, confirming that ableism is still a challenge that persons with disabilities face in general. The SAMA’s efforts to improve the knowledge and attitudes of doctors about disability should be intentional in manifesting empathy and respect to ensure appropriate accommodation is provided to their patients and colleagues.
